# 17-DMAG inhibits the multiplication of several *Babesia* species and *Theileria equi* on *in vitro* cultures, and *Babesia microti* in mice

**DOI:** 10.1016/j.ijpddr.2018.02.005

**Published:** 2018-03-01

**Authors:** Azirwan Guswanto, Arifin Budiman Nugraha, Bumduuren Tuvshintulga, Dickson Stuart Tayebwa, Mohamed Abdo Rizk, Gaber El-Saber Batiha, Sambuu Gantuya, Thillaiampalam Sivakumar, Naoaki Yokoyama, Ikuo Igarashi

**Affiliations:** a National Research Center for Protozoan Diseases, Obihiro University of Agriculture and Veterinary Medicine, Nishi 2-13 Inada-cho, Obihiro 080-8555, Japan; b Balai Veteriner Subang (DIC Subang), Jl. Terusan Garuda 33/11 Blok Werasari Dangdeur, Subang, Jawa Barat 41212, Indonesia; c Department of Animal Infectious Diseases and Veterinary Public Health, Faculty of Veterinary Medicine, Bogor Agricultural University, Jl. Agatis, Kampus IPB Dramaga, Bogor, Indonesia; d Department of Internal Medicine and Infectious Diseases, Faculty of Veterinary Medicine, Mansoura University, Mansoura, 35516, Egypt; e Department of Pharmacology and Therapeutics, Faculty of Veterinary Medicine, Damanhour University, Al-Beheira, 22511, Egypt

**Keywords:** 17-DMAG, *Babesia*, Chemotherapeutic, Hsp90 inhibitor, *Theileria*, 17-DMAG, 17-dimethylaminoethylamino-17-demethoxygeldanamycin, 17-AAG, 17-N-allylamino-17-demethoxygeldanamycin (tanespimycin), AV, atovaquone, BW, body weight, CI, combination index values, DA, diminazene aceturate, IC_50_, half maximum inhibition concentration, Hsp90, heat shock protein 90, MDBK, Madin-Darby Bovine Kidney cell line, NIH/3T3, mouse embryonic fibroblast cell line, p.i, post infection, RBC, red blood cells

## Abstract

Heat shock protein 90 (Hsp90) is a chaperone protein that stabilizes cells during stress or non-stress responses. Previous reports have shown that Hsp90 is a potential drug target to suppress the multiplication of several protozoan parasites. In this study, 17-dimethylaminoethylamino-17-demethoxygeldanamycin (17-DMAG), an Hsp90 inhibitor, was evaluated for its inhibitory effect on five *in vitro* cultures of *Babesia* and *Theileria* species, including *B. bovis*, *B. bigemina*, *B. divergens*, *B. caballi*, and *T. equi*, and on the multiplication of a *B. microti*–infected mouse model. 17-DMAG showed the inhibitory effect in all of the species tested. The half maximum inhibition concentration (IC_50_) of 17-DMAG on *B. bovis*, *B. bigemina*, *B. divergens*, *B. caballi*, and *T. equi* was 77.6 ± 2.9, 62.4 ± 1.9, 183.8 ± 3.2, 88.5 ± 9.6, and 307.7 ± 7.2 nM, respectively. The toxicity assay on MDBK and NIH/3T3 cell lines showed that 17-DMAG affected the viability of cells with an IC_50_ of 15.5 ± 4 and 8.8 ± 2 μM, respectively. Since the IC_50_s were much lower on the parasites than on the host cell lines, the selectivity index were high for all tested species. Furthermore, the two-drug combination of 17-DMAG with diminazene aceturate (DA) and atovaquone (AV) showed synergism or addition on *in vitro* cultures of *Babesia* and *Theileria* parasites. In the mouse model, 17-DMAG at a concentration of 30 mg/kg BW effectively inhibited the multiplication of *B. microti*. Moreover, if combined with DA or AV, 17-DMAG showed a comparable inhibition at the half dose. Taken together, these results indicate that 17-DMAG is a potent drug for treating piroplamosis. The data warrant further evaluation of 17-DMAG as an antibabesial drug and as an option in combination with atovaquone for the treatment of human babesiosis.

## Highlights

• 17-DMAG inhibits the *in vitro* multiplication of *Babesia* and *Theileria* parasites.
• Combination of 17-DMAG with diminazene aceturate or atovaquone were also effective.
• 17-DMAG also inhibits the multiplication of *B. microti* in mice.
• 17-DMAG is a new treatment option for babesiosis in animal and human.

## Graphical abstract

**Figure:**
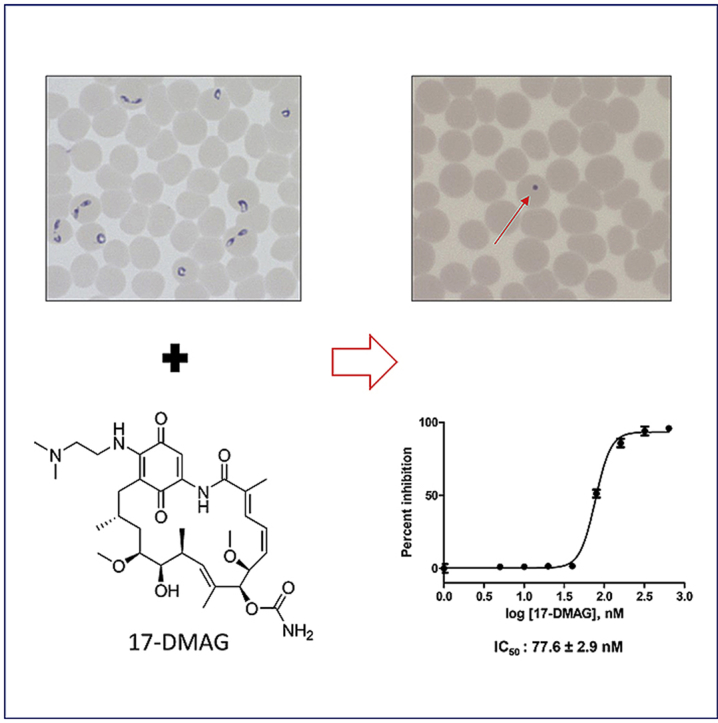

## 1 Introduction

The chemoprophylaxis agents against babesiosis in the livestock industries remain inadequate. After decades of use, diminazene aceturate and imidocarb dipropionate are still the first choices for the treatment of animals (Suarez and Noh, 2011; Oliveira and de Freitas, 2015). However, several problems such as the development of a resistant parasite, toxicity, drug residues, and withdrawal issues constrain the use of these drugs in many countries (Kuttler, 1980; Coldham et al., 1995; Baldissera et al., 2016). Furthermore, they are not approved for human medicine. The preferable treatment of babesiosis in humans is the combination of atovaquone with azithromycin due to their low side effects (Krause et al., 2000). Yet *Plasmodium falciparum* rapidly developed resistance when atovaquone was used as a single drug (Korsinczky et al., 2000). Another report showed the relapse of *Babesia gibsoni* due to the change of amino acid in the mitochondrial cytochrome B that led to a reduction in the efficacy of atovaquone (Matsuu et al., 2006). Therefore, continuous efforts to discover and develop new effective drugs against babesiosis are very important.

Heat shock proteins (Hsps), which are present in most eukaryotes and prokaryotes, are involved in stabilizing their client proteins to enable appropriate functions during a stress or non-stress response (Kumar et al., 1990; Ruef et al., 2000). Heat shock protein 90 (Hsp90), one of the Hsp classes, is conserved among organisms (Chen et al., 2006). Due to its important role in supporting the cellular mechanism, this protein has been targeted for combating cancer cells in humans (Kim et al., 2009). In protozoan parasites, the protein has been reported to regulate the cellular processes in zoonotic protozoan parasites such as *Plasmodium*, *Toxoplasma*, *Trypanosoma,* and *Leishmania* (Banumathy et al., 2003; Angel et al., 2013). Furthermore, several studies have shown the effectiveness of Hsp90 as drug target for infectious diseases (Pizarro et al., 2013; Gillan et al., 2014).

The first inhibitor found specifically to bind Hsp90 was geldanamycin, which was isolated from the bacterium *Streptomyces hygroscopicus*. The inhibition of geldanamycin on Hsp90 led to the degradation of its client proteins. However, geldanamycin has poor solubility, induces liver damage, and is toxic to the erythrocyte (Jilani et al., 2013). Many analogs have been developed on the basis of geldanamycin, such as 17-AAG (tanespimycin, 17-N-allylamino-17-demethoxygeldanamycin) and 17-DMAG (alvespimycin, 17-dimethylaminoethylamino-17-demethoxy geldanamycin). 17-AAG has less hepatotoxicity than geldanamycin. It has been evaluated in cancer studies and, in protozoan parasites, showed growth inhibition on a rodent model of malaria (Pallavi et al., 2010). However, like geldanamycin, 17-AAG also showed poor solubility, and its clinical trial in cancer has been halted. On the other hand, 17-DMAG is a water soluble. It has been evaluated on the *in vitro* culture and mouse model of *Trypanosoma brucei*. The result showed a very low IC_50_ on the parasite and a high selectivity index over mammalian cells, resulting in a great potency of this compound as an antitrypanosomal agent (Meyer and Shapiro, 2013).

This study aimed to evaluate the inhibition of 17-DMAG on the multiplication of bovine *Babesia* (*B. bovis* and *B. bigemina*), bovine *Babesia* that is also known to infect humans (*B. divergens*), equine piroplasms parasites (*B. caballi* and *T. equi*), and rodent *Babesia* that also infects humans (*B. microti*). The evaluations were performed using *in vitro* cultures of bovine and equine species and, for *B. microti*, using a mouse model. The cytotoxicity of 17-DMAG was also evaluated using mammalian cells including Madin-Darby Bovine Kidney (MDBK) and mouse embryonic fibroblast (NIH/3T3) cell lines.

## 2 Materials and methods

### 2.1 Parasites

*Babesi*a parasite cultures were maintained using a microaerophilic stationary-phase culture system (Levy and Ristic, 1980; Avarzed et al., 1997; Igarashi et al., 1998). Briefly, bovine *Babesia* parasites were grown in bovine red blood cells (RBC) in the specific complete medium for each species. The medium for *B. bovis* (Texas strain) contained GIT medium supplemented with 10% bovine serum, while the medium for *B. bigemina* (Argentina strain) and *B. divergens* (Germany strain) was Medium 199 and RPMI 1640 medium, respectively, supplemented with 40% bovine serum (Rizk et al., 2016). *B. caballi* (USDA strain) was grown using equine RBC in GIT medium supplemented with 10% equine serum. *T. equi* (USDA strain) was grown in equine RBC in M199 medium supplemented with 40% equine serum and hypoxanthine (MP Biomedicals, USA) at a final concentration of 13.6 μg/ml. All of the media included 60 U/ml penicillin G, 60 μg/ml streptomycin, and 0.15 μg/ml amphotericin B (Sigma-Aldrich, USA). The cultures were incubated at 37 °C in a humidified chamber with an atmosphere of 5% CO2, 5% O2, and 90% N2.

*B. microti* (Munich strain) was recovered from −80 °C stock in two 6-week female Balb/c mice (Clea, Japan). The parasitemia was monitored every 2 days. After parasitemia reached approximately 30%, mice were euthanized, and blood was collected by cardiac puncture to initiate the *in vivo* experiment (Goo et al., 2010). The animal experiment was conducted in accordance with The Regulations for Animal Experiments of Obihiro University of Agriculture and Veterinary Medicine, Japan (Accession numbers 28-111-2, 28-110, and 1417-2).

### 2.2 Reagents and chemicals

17-DMAG (Focus Biomolecules, USA), diminazene aceturate (DA, Sigma-Aldrich, Japan), and atovaquone (AV, Sigma-Aldrich, Japan) were diluted in DMSO to make a 10 mM stock solution, which was stored at −30 °C until use in the *in vitro* experiment. For the *in vivo* experiment, each compound was weighed according to the average mouse weight and dissolved with a suitable solvent before use. A lysis buffer containing tris-HCl (130 mM; pH 7.5), EDTA (10 mM), saponin (0.016%; w/v), and Triton X-100 (1.6% v/v) was prepared, filtered through 0.22 μm of polyethersulfone, and stored at 4 °C. Prior to fluorescence measurement, the lysis buffer was mixed with 0.2 μl/ml SYBR Green I (10,000x, Lonza, USA).

### 2.3 Effect of 17-DMAG on the erythrocytes of bovines and equines, and on uninfected mice

Prior to the subculture of parasites, bovine and equine RBC were incubated with 1 μM of 17-DMAG for 3 h. The RBC were washed three times with PBS and mixed with *B. bovis* and *T. equi* parasitized RBC to obtain 1% parasitemia (infected RBC). Subsequently, 100 μl of infected RBC was added to 900 μl of complete medium in a 24-well plate. The untreated erythrocytes were used as a control. The parasitemia was monitored daily via microscopy observation of a Giemsa-stained blood smear for 4 days. Furthermore, the effect of 17-DMAG on mice was evaluated using 8-week-old BALB/c mice. Groups 1 and 2, consisting of three mice each, received intraperitoneal injections of 17-DMAG at a concentration of 15 and 30 mg/kg BW, respectively, for 5 days. One group (three mice) remained as an untreated control. The changes in body weight and hematology profiles, including RBC, hematocrit, and hemoglobin, were observed for 20 days.

### 2.4 Inhibition assay of 17-DMAG and combination with diminazene aceturate and atovaquone *in vitro*

The assays were conducted in accordance with the protocol described previously with modification (Guswanto et al., 2014). Briefly, the cultures of *B. bovis*, *B. bigemina*, *B. divergens*, *B. caballi*, and *T. equi* with a parasitemia of 8%, 3%, 10%, 4%, and 9%, respectively, were harvested and adjusted to 1% parasitemia with fresh RBC before the inhibition assay. The 60 inner wells of a 96-well plate were used in the assay, while the peripheral wells were filled with sterile distillate water to reduce evaporation during incubation. To each well in triplicate, 2.5 μl of infected RBC (5 μl for *B. divergens*, *B. caballi*, and *T. equi*) was added and mixed with a culture medium containing the drug to a total volume of 100 μl. The stock solution of 17-DMAG, DA, and AV was dissolved in a culture medium at various concentrations using two-fold dilution. A culture medium containing DMSO at a final concentration of 0.2% was added to the well with infected RBC as a positive control and to the well with uninfected RBC as a negative control. Furthermore, a drug combination assay was conducted in parallel with the single drug assay at the constant ratio in accordance with the Chou–Talalay method (Chou, 2006). In the same plate with a single drug inhibition assay, a two-drug combination (DA+17-DMAG, DA + AV, and 17-DMAG + AV) at concentrations of 0.25 x IC_50_, 0.5 x IC_50_, IC_50_, 2 x IC_50_, and 4 x IC_50_ was added to the well containing infected RBC in triplicate. The plate was incubated in a humidified incubator with 5% CO_2_, 5% O_2_, and 90% N_2_. After 96 h, 100 μl of a lysis buffer containing SYBR Green I was added to each well. The plate was wrapped with aluminum foil to avoid direct light exposure and incubated at room temperature. After incubation for 6 h, fluorescence values were measured using a fluorescence spectrophotometer (Fluoroskan Ascent, Thermo Fisher Scientific, USA) with the excitation and emission wavelength at 485 nm and 518 nm, respectively. The fluorescence data were subtracted from the negative control and used to calculate the IC_50_.

### 2.5 Cell cultures

MDBK and NIH/3T3 cells were maintained in 75 cm^2^ culture flasks with MEM medium (Gibco, Life Technologies, USA) and DMEM (Gibco, Life Technologies, USA), respectively, and cultured in a humidified incubator at 37 °C with 5% CO_2_. Each medium was supplemented with 10% fetal bovine serum, 0.5% penicillin/streptomycin (Gibco, Life Technologies, USA), and an additional 1% glutamine. The medium was changed every 3–4 days and incubated until approximately 80% confluent. The cells were free from mycoplasma contamination after being checked by staining with DAPI (Sigma-Aldrich, USA). The detachment of cells from the culture flask was done using TrypLE™ Express (Gibco, Life Technologies, USA) after washing two times with Dulbecco's phosphate-buffered saline (DPBS). Subsequently, the counting of viable cells was carried out using a Neubauer improved C-Chip (NanoEntek Inc., Korea) after staining with 0.4% trypan blue solution.

### 2.6 Cytotoxicity assay of 17-DMAG, diminazene aceturate, and atovaquone on MDBK and NIH/3T3 cell lines

The drug-exposure viability assay was performed in accordance with the recommendation of the Cell Counting Kit-8 (CCK-8, Dojindo, Japan). Briefly, the assay was carried out using a 96-well plate at 37 °C in a humidified incubator with 5% CO_2_. One hundred microliters of cells at a density of 5 × 10^4^ cells/ml was seeded per well and allowed to attach to the plate for 24 h. Ten microliters of twofold drug dilutions was added to each well to a final concentration of 0.312–40 μM in triplicate. The wells with only a culture medium were used as blanks, while the wells containing cells in a medium with 0.4% DMSO were used as negative control, representing the highest concentration of drug solvent. The exposure of drugs was carried out for 24 h, followed by the addition of 10 μl of CCK-8. The plate was further incubated for 3 h, and the absorbance was measured at 450 nm using a microplate reader.

### 2.7 Inhibition assay using a *B. microti*–infected mouse model

The inhibitory effect of 17-DMAG was also determined using a *B. microti* mouse model according to the previous protocol (Goo et al., 2010). Briefly, 35 female BALB/c mice aged 8 weeks received an intraperitoneal injection of 1 × 10^7^ of *B. microti*–infected RBC and were divided into seven groups. Another group consisting of five mice was kept uninfected and untreated as a control. The multiplication of *B. microti* was observed from blood smears after staining with Giemsa using a light microscope. The drug treatment was initiated to all infected mice on day five, after the parasitemia reached approximately 1%, and continue to the total of five consecutive days. Group 1 was treated with 4% DMSO in saline water as a control intraperitoneally. Groups 2–4 received an intraperitoneal injection of 30 mg/kg BW of 17-DMAG, an injection of 25 mg/kg BW of DA, and oral administration of 20 mg/kg BW of AV, respectively. Groups 5–7 were treated with a combination of 17-DMAG + DA (15 + 15 mg/kg BW), 17-DMAG + AV (15 + 10 mg/kg BW), and DA + AV (15 + 10 mg/kg BW), respectively, via a route similar to that for the single drug. The parasitemia was estimated from Giemsa-stained blood smears using microscopy in approximately 5000 RBC. Furthermore, the hematology profiles including RBC, hemoglobin, and hematocrit were measured from 10 μl of mouse blood using an automatic hematology analyzer (Celltac α MEK-6450, Nihon Kohden, Tokyo, Japan). The rate of parasitemia, hematology profiles, and mouse body weight was monitored for 60 days. The experiment was repeated once so each group contained 10 mice.

### 2.8 Statistical analysis

The IC_50_ of 17-DMAG, DA, and AV was calculated from the percentage of inhibition on the *in vitro* multiplication of all tested species using non-linear regression (curve fit), available in GraphPad Prism (GraphPad Software Inc., USA). Combination index (CI) values for the drug combination were calculated using CompuSyn software (Chou, 2006). Since the chemotherapy effect is preferable at the highest inhibition, the degree of synergism was determined as the weighted average of CI values using a formula ((1 x IC_50_) + (2 x IC_75_) + (3 x IC_90_) + (4 x IC_95_))/10 (Chou, 2006). The differences among groups regarding the parasitemia, hematology profiles, and body weight in the *B. microti*–infected mouse model were analyzed using Student's *t*-test, available in GraphPad Prism software. The difference was considered significant if *P* < 0.05.

## 3 Results

Original data of this study including percent inhibition of drug on each parasite, drug combination data, and *in vivo* observations during drug treatment including parasitemia, mice body weight, and hematology profiles are available at Mendeley Data (https://doi.org/10.17632/6sdnnh9hng.1).

### 3.1 17-DMAG inhibition of *Babesia* and *Theileria* parasite multiplication *in vitro*

**Fig. 1:**
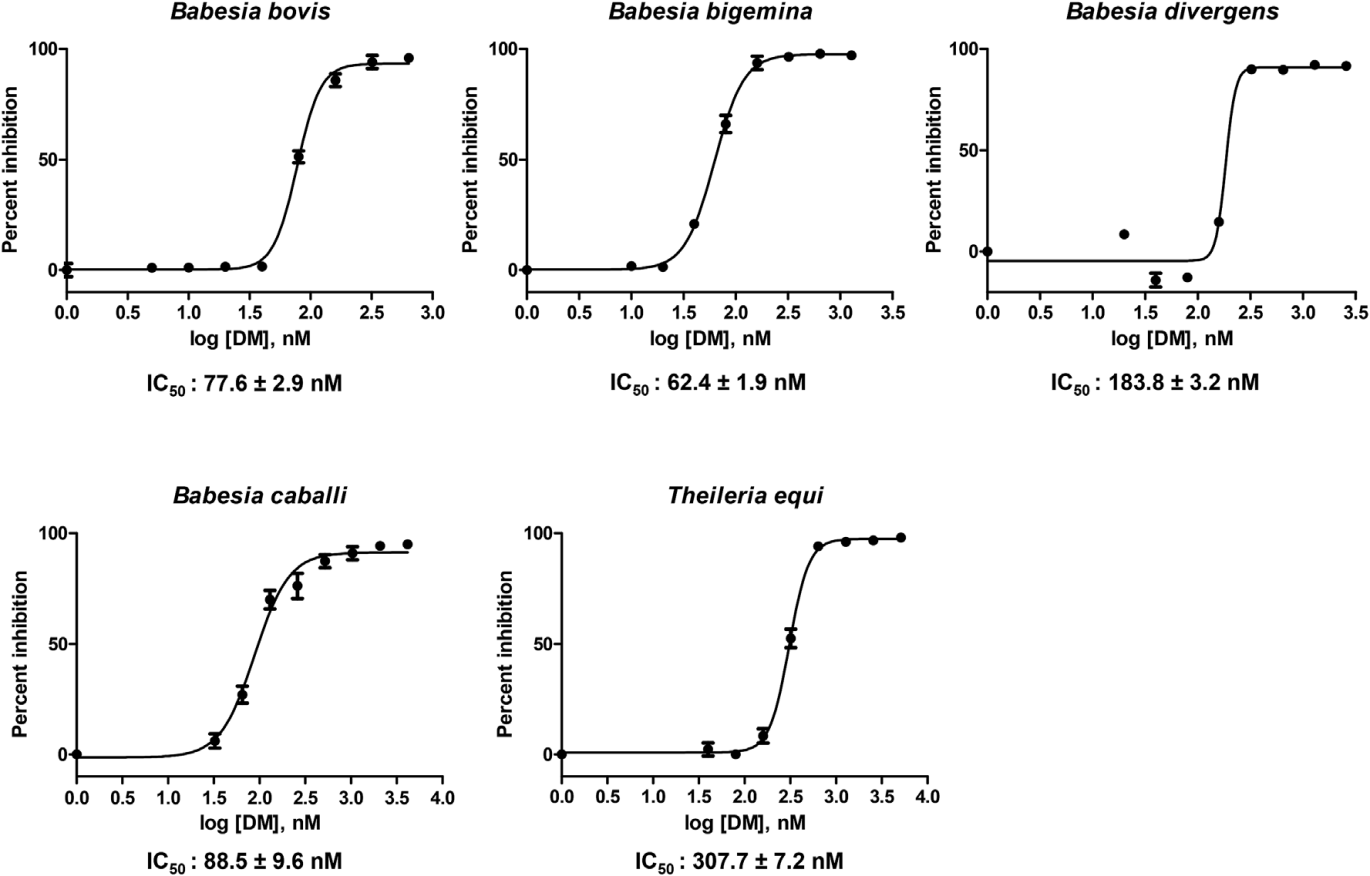
**Dose response curve of 17-DMAG on the *in vitro* cultures.** The parasitemia of *B. bovis*, *B. bigemina*, *B. divergens*, *B. caballi*, and *T. equi* after exposure to various concentrations of 17-DMAG for 96 h as determined by SG-based fluorescence assay. The percentage of inhibition is expressed as the percentage of inhibited parasites compared with the positive control (untreated wells) after subtracting the negative control (uninfected RBC). The data were the mean and S.D. from triplicate experiments.

The preliminary evaluation of 17-DMAG was conducted to determine its direct effect on host RBC. Bovine and equine RBC were incubated with 17-DMAG (1 μM) for 3 h prior to the subculture of *B. bovis* and *T. equi*. Microscopy observation and parasitemia counting showed that the multiplication of *B. bovis* and *T. equi* did not significantly differ between 17-DMAG-treated RBC and normal RBC for either species. Evaluation was also conducted using mice. Mouse body weight was reduced slightly in the group of 30 mg/kg BW on day 10 post treatment but was not significantly different from that of the control group, while the group of 15 mg/kg BW did not show any effect (Supplementary Fig. S1). For the multiplication inhibitory effect, the assay was conducted on five species: *B. bovis*, *B. bigemina*, *B. divergens*, *B. caballi*, and *T. equi*. 17-DMAG inhibited the multiplication of all species tested (Fig. 1). The IC_50_ of 17-DMAG on *B. bovis*, *B. bigemina*, *B. divergens*, *B. caballi*, and *T. equi* was 77.6 ± 2.9, 62.4 ± 1.9, 183.8 ± 3.2, 88.5 ± 9.6, and 307.7 ± 7.2 nM, respectively (Table 1). Compared with other drugs tested in this study, 17-DMAG was more effective than DA for *B. bovis*, *B. bigemina*, and *B. divergens* (Supplementary Fig. S2). It was also more effective for *B. bigemina* and *B. caballi* compared with the IC_50_ of AV (Supplementary Fig. S3).

**Table 1:** The IC_50_ and selectivity index of 17-DMAG, diminazene aceturate, and atovaquone.

Drug	Parasites	IC_50_ (nM)^a^	IC_50_ (μM)^b^		Selectivity index^c^	
			NIH/3T3	MDBK	NIH/3T3	MDBK
17-DMAG	*B. bovis*	77.6 ± 2.9	15.5 ± 4.0	8.8 ± 2.0	200	113
	*B. bigemina*	62.4 ± 1.9			248	141
	*B. divergens*	183.8 ± 3.2			84	48
	*B. caballi*	88.5 ± 9.6			175	99
	*T. equi*	307.7 ± 7.2			50	29

DA	*B. bovis*	189.8 ± 42.1	>40	>40	>211	>211
	*B. bigemina*	1852 ± 104			>22	>22
	*B. divergens*	377.5 ± 39.3			>106	>106
	*B. caballi*	13.4 ± 3.6			>2985	>2985
	*T. equi*	59. ± 8.5			>668	>668

AV	*B. bovis*	39.7 ± 2.4	>40	>40	>1008	>1008
	*B. bigemina*	706.1 ± 38.7			>57	>57
	*B. divergens*	13.4 ± 1.4			>2985	>2985
	*B. caballi*	101.9 ± 14.1			>393	>393
	*T. equi*	95.0 ± 65			>421	>421

a Half maximum inhibition concentration of each drug on the *in vitro* culture of parasites. The value was determined from the dose-response curve using non-linear regression (curve fit analysis). The values are the mean and standard deviation of triplicate experiments.

b Half maximum effective concentration of each drug on cell lines. The values were determined from the dose-response curve using non-linear regression (curve fit analysis). The values are the mean and standard deviation of triplicate experiments.

c Ratio of the IC_50_ on cell lines to the IC_50_ on each species. High numbers are favorable.

### 3.2 Combination of 17-DMAG with diminazene aceturate and atovaquone on *in vitro* cultures

**Fig. 2:**
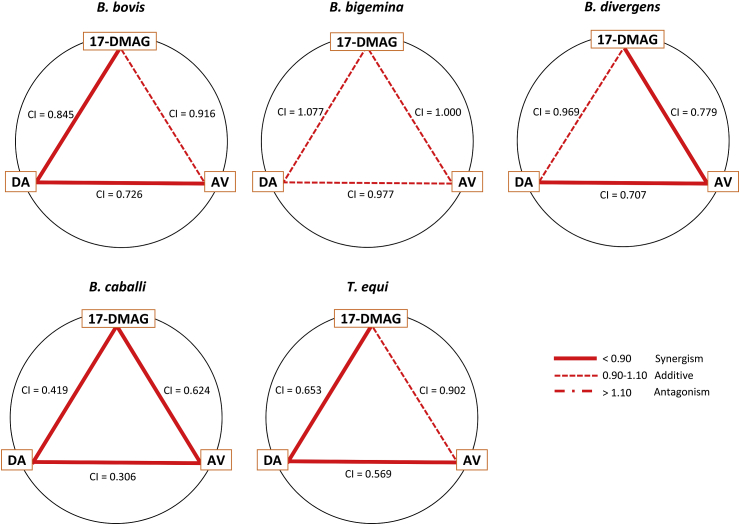
**Polygonogram of the degree of synergism.** The polygonogram of synergism between 17-DMAG, diminazene aceturate (DA), and atovaquone (AV) based on the weighted average of combination index values (CIs) at IC_50_, IC_75_, IC_90_, and IC_95_ using the formula ((1 x IC_50_) + (2 x IC_75_) + (3 x IC_90_) + (4 x IC_95_))/10 (Chou, 2006). The degree of synergism was determined based on the following CI value: < 0.90 (synergism), 0.90–1.10 (additive), and >1.10 (antagonism).

The drug combination analysis was to determine whether the combined treatments are synergism (give a greater effect), additive (similar effect), or antagonism (reduce or block the effect). Five dilutions of 17-DMAG, as recommended in the Chou–Talalay method (Chou, 2006), were combined at a constant ratio of DA or AV. The percentage of inhibition of the single drug and each combination was analyzed using CompuSyn software to generate the combination index (CI) value at IC_50_, IC_75_, IC_90_, and IC_95_ (Table 2). The weighted average CI values were visualized using a polygonogram (Fig. 2). The drug combination was considered as synergism if the value was less than 0.90, additive if the value was at a range of 0.90–1.10, and antagonism if the value was more than 1.10. Our results showed that the combination of 17-DMAG with DA was synergistic on *B. bovis*, *B. caballi*, and *T. equi*, while that with AV was synergistic on *B. divergens* and *B. caballi*. Other species showed additive effects, and none of the combinations showed antagonism.

**Table 2:** Combination index (CI) value of a two-drug combination between 17-DMAG, diminazene aceturate, and atovaquone.

Parasites	Drug combinations^a^	CI value at				Weighted average CI values^b^	Degree of synergism^c^
		IC_50_	IC^c^_5_	IC_90_	IC_95_		
*B. bovis*	17-DMAG + DA	0.868	0.853	0.842	0.838	0.845	Synergism
	17-DMAG + AV	1.255	1.051	0.884	0.787	0.916	Additive
	DA + AV	0.725	0.725	0.726	0.727	0.726	Synergism

*B. bigemina*	17-DMAG + DA	1.353	1.194	1.054	0.968	1.077	Additive
	17-DMAG + AV	1.181	1.073	0.983	0.930	1.000	Additive
	DA + AV	1.054	1.006	0.969	0.949	0.977	Additive

*B. divergens*	17-DMAG + DA	1.850	1.269	0.870	0.673	0.969	Additive
	17-DMAG + AV	2.072	1.111	0.613	0.416	0.779	Synergism
	DA + AV	1.217	0.876	0.650	0.539	0.707	Synergism

*B. caballi*	17-DMAG + DA	0.693	0.503	0.387	0.332	0.419	Synergism
	17-DMAG + AV	1.085	0.789	0.574	0.463	0.624	Synergism
	DA + AV	0.290	0.288	0.303	0.321	0.306	Synergism

*T. equi*	17-DMAG + DA	1.168	0.786	0.591	0.503	0.653	Synergism
	17-DMAG + AV	1.224	1.033	0.872	0.778	0.902	Additive
	DA + AV	0.925	0.652	0.526	0.472	0.569	Synergism

CI value, combination index value; IC_50_, 50% inhibition concentration; DA, diminazene aceturate; AV, atovaquone.

a Two-drug combination between 17-DMAG, diminazene aceturate, and atovaquone at a concentration of approximately 0.25 x IC_50_, 0.5 x IC_50_, IC_50_, 2 x IC_50_, and 4 x IC_50_ (constant ratio).

b The higher inhibition is preferable, thus the weighted average CI value was calculated with the formula ((1 x IC_50_) + (2 x IC_75_) + (3 x IC_90_) + (4 x IC_95_))/10.

c The degree of synergism was determined based on the following CI value: < 0.90 (synergism), 0.90–1.10 (additive), and >1.10 (antagonism).

### 3.3 Toxicity of 17-DMAG on MDBK and NIH/3T3 cell lines

17-DMAG showed an effective inhibitory effect similar to that with currently used drugs, DA and AV, on the *in vitro* culture of *Babesia* and *Theileria* parasites. The effect of 17-DMAG on the host was evaluated using MDBK and NIH/3T3 cell lines. The IC_50_ of 17-DMAG on MDBK cells was 8.8 ± 2 μM, while that on NIH/3T3 cells was 15.5 ± 4 μM. The result showed that 17-DMAG is more toxic than DA and AV. In a separate assay, DA and AV at a concentration of 40 μM did not show any inhibition on MDBK and NIH/3T3 cell viability. The selectivity index, defined as the ratio of cell line IC_50_ to the parasite IC_50_, is shown in Table 1. For the NIH/3T3 cells, the highest selectivity index of 17-DMAG was found to be 248 times higher than the IC_50_ on *B. bigemina*, while the lowest was on *T. equi* with a selectivity index of 50. The highest and lowest selectivity index for MDBK cells was also determined on *B. bigemina* and *T. equi*, respectively.

### 3.4 17-DMAG inhibition of *B. microti* multiplication using a mouse model

**Fig. 3:**
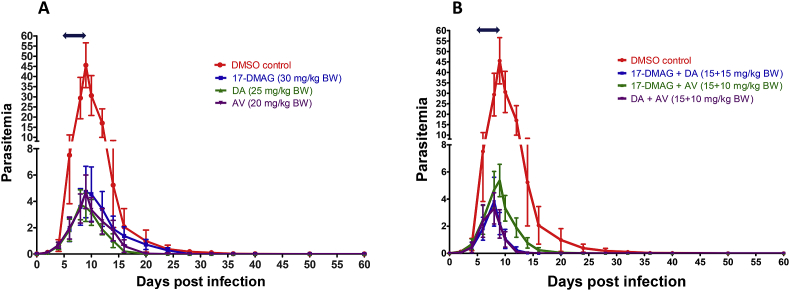
**Effect of drug treatment on the growth of *Babesia microti* in mice.** (A) Parasitemia after treatment with 17-DMAG, diminazene aceturate (DA), and atovaquone (AV) at a concentration of 30, 25, and 20 mg/kg body weight, respectively. (B) Parasitemia after treatment with the combination of 17-DMAG and DA at 15 mg/kg BW each, 17-DMAG with AV at 15 and 10 mg/kg BW, respectively, and a combination of DA and AV at 15 and 10 mg/kg BW, respectively. Drug administrations were started from day 4 until day 8 post infection (left right arrow). The data were the mean and S.D. from two separate experiments (in total of 10 mice per group).

**Fig. 4:**
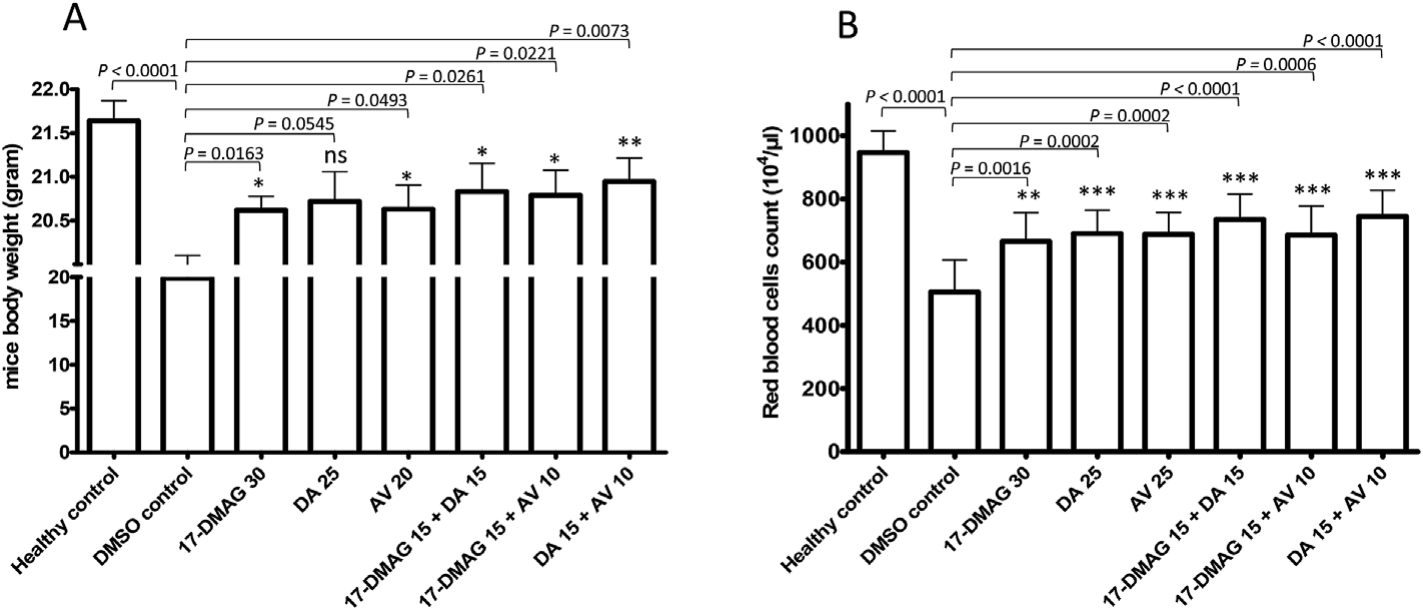
**Body weight and hematology profiles of mice on day 10 post infection.** The observation of mouse body weight (A) and red blood cell count (B) of mice on day 10 post infection. All data were the mean and S.D. from 10 mice in each group as a comparison with DMSO control and healthy control. The significant differences are marked with * if *P* is less than 0.05, ** if *P* is less than 0.01, and *** if *P* is less than 0.001.

The inhibitory effect of 17-DMAG was further evaluated on the multiplication of *B. microti*. The experiment was carried out using a mouse model, since the *in vitro* culture of *B. microti* is not well established. The mice were divided into eight groups (five mice/group), corresponding to single drug treatment, controls, and combinations of 17-DMAG, DA, and AV. The drugs were combined at approximately half the concentration of single drug treatment. The experiment was conducted two times, with each group containing 10 mice. In the DMSO control group, the multiplication of *B. microti* increased significantly and reached the highest parasitemia at 45.6 ± 10.5% on day 9 post infection (p.i). The parasitemia decreased gradually on the following days. On day 60, *B. microti* was still detected in four mice. *B. microti* in the 17-DMAG 30 mg/kg BW group reached peak parasitemia (4.7 ± 2.0%) on day 10 p.i., and no parasite was detected via microscopy starting on day 36 p.i. Similar inhibition was observed on the DA (25 mg/kg BW) and AV (20 mg/kg BW) groups, but the parasite had disappeared from blood smears by day 28 (Fig. 3A). However, 17-DMAG seems to be a slow-acting drug as compared with DA and AV, since it postponed the peak of parasitemia even though it had an inhibitory effect. The combination of 17-DMAG (15 mg/kg BW) with DA (15 mg/kg BW) or AV (10 mg/kg BW) also showed an inhibitory effect comparable to that with a single treatment at a higher dose (Fig. 3B). However, the parasite clearance was faster than that of single drug treatments. The parasite was not detected in the blood smear on the day 24 p.i in the 17-DMAG + DA (15 + 15 mg/kg BW) group and in the DA + AV (15 + 10 mg/kg BW) group. In the DM + AV (15 + 10 mg/kg BW) group, the parasite clearance was observed on day 28 p.i. Furthermore, infection with *B. microti* reduces the hematocrit, RBC, and hemoglobin concentration in mouse blood, as observed in the DMSO control group on day 10 p.i. (*P* < 0.0001). Significant differences in mouse body weight were observed between the DMSO control group and all drug-treated groups except for the DA (25 mg/kg BW) group (Fig. 4A). The RBC (Fig. 4B), hemoglobin (Fig. S4.A), and hematocrit counts (Fig. S4.B) were also determined to be significantly different between the DMSO control group and all drug-treated groups (*P* < 0.0001).

## 4 Discussion

Treatment of babesiosis remains a significant challenge, especially in the livestock industries. Although diminazene aceturate and imidocarb dipropionate are still effective, current reports showed the development of resistance in *Trypanosoma evansi* in cattle and a relapse of *B. gibsoni* in dogs (Witola et al., 2005; Rajapakshage et al., 2012; Baldissera et al., 2016). Many inhibitors have been reported to be effective against *in vitro* or *in vivo* multiplication of *Babesia* and *Theileria* parasites; however, all of them are in research and still far from clinical trial (Mosqueda et al., 2012). Furthermore, the emergence of human babesiosis brings a new urgency to find an effective inhibitor of babesiosis. Atovaquone, which is effective on *Plasmodium*, was also found to inhibit the multiplication of *Babesia* parasites (Vial and Gorenflot, 2006). Since this drug was reported to be less toxic to host cells, its combination with azithromycin is currently suggested for the treatment of human babesiosis. However, the development of a resistant parasite limited the use of atovaquone. Therefore, the evaluation of a new potent compound against *Babesia* and *Theileria* parasites will provide more options for treatment of the disease both in humans and domestic animals.

This study showed that 17-DMAG inhibits the multiplication of several *Babesia* species and *Theileria equi in vitro*. Its ability to bind Hsp90 causes a significant reduction in parasite survival. Hsp90 is conserved in most organisms, including *Babesia* and *Theileria* parasites. There are some isoforms of Hsp90 in *B. bovis* based on reported full genome sequences (Brayton et al., 2007). These proteins protect the organism during physical and non-physical stress and enable the proper synthesis and stabilization of its client proteins. Targeting Hsp90 is more relevant in cancer treatment because many cancer Hsp90–related client proteins are involved in the progression of cancer cells (Miyata et al., 2013). However, recent studies showed that the inhibition of parasite Hsp90 could disrupt the stage differentiation of parasites such as *Toxoplasma gondii* (Echeverria et al., 2005), *Plasmodium falciparum* (Banumathy et al., 2003), *Eimeria tenella* (Péroval et al., 2006), *Trypanosoma brucei* (Meyer and Shapiro, 2013), and *Theileria annulata* (Kinnaird et al., 2017) that lead to the halt of their growth. In agreement with those previous studies, we hypothesized that 17-DMAG might be attached to the binding domain of *Babesia* and *Theileria* parasite Hsp90, causing insufficient binding of client proteins to Hsp90 and disrupting the normal multiplication of parasites.

Hsp90 is also conserved in the host cells, which means that the binding of the inhibitor to the host's Hsp90 is possible. For this reason, the inhibitor should specifically bind to the parasite Hsp90 to avoid toxicity to the host. The basis of Hsp90 as a drug target in *Plasmodium falciparum* was the increasing ATPase activities in this species compared with human Hsp90. The high stress conditions during parasite invasion of host cells might increase the activity of their Hsp90 and evolved significantly (Pallavi et al., 2010). Our study of MDBK and NIH/3T3 cells showed that the selectivity index of 17-DMAG was quite high among the species tested. This means that 17-DMAG was more likely to bind to the *Babesia* and *Theileria* Hsp90 than to the host Hsp90. Similar to *Plasmodium falciparum*, *Babesia* and *Theileria* parasites experience high stress during their invasion of host cells. Further study of the biochemical characterization of Hsp90 in *Babesia* and *Theileria* is required to better understand the potency of this protein as a drug target.

The potency of 17-DMAG as an inhibitor was further evaluated in a combination study using DA and AV. The cytotoxicity assay showed that 17-DMAG was more toxic than DA and AV. The combination study aimed to enhance the potency of 17-DMAG while reducing the dose that led to reduced toxicity. Our results demonstrated that the combination of 17-DMAG and DA or AV against all species was range from synergism to additive. Therefore, the combination of 17-DMAG and DA might have potential as a novel regime for treating piroplasmosis in a wide range of animals. Furthermore, 17-DMAG might be used as a treatment option in combination with AV for the treatment of human babesiosis.

The potent effect of 17-DMAG combined with DA or AV was also observed on the multiplication of *B. microti* in a mouse model. The combination of 17-DMAG with DA or AV at a half dose showed a multiplication inhibition comparable to that of those single drugs at full dose. Hematology profiles such as RBC count, hemoglobin, and hematocrit were also improved compared with the DMSO control group, as were parasitemia and mouse body weight. Taken together, these results indicate that 17-DMAG is a potent antibabesial and antitheilerial drug, as confirmed by the inhibition of five species, *B. bovis*, *B. bigemina*, *B. divergens*, *B. caballi*, and *T. equi* on *in vitro* cultures and *B. microti in vivo*. Even though 17-DMAG showed toxicity in the host cells, its combination with diminazene aceturate and atovaquone could improve the effect while reducing the toxicity.

## References

[bib1] Angel S.O., Matrajt M., Echeverria P.C. (2013). A review of recent patents on the protozoan parasite Hsp90 as a drug target. Recent Pat. Biotechnol..

[bib2] Avarzed A., Igarashi I., Kanemaru T., Hirumi K., Omata Y., Saito A., Oyamada T., Nagasawa H., Toyoda Y., Suzuki N. (1997). Improved *in vitro* cultivation of *Babesia caballi*. J. Vet. Med. Sci..

[bib3] Baldissera M.D., Grando T.H., Souza C.F., Cossetin L.F., Sagrillo M.R., Nascimento K., da Silva A.P., Dalla Lana D.F., Da Silva A.S., Stefani L.M., Monteiro S.G. (2016). Nerolidol nanospheres increases its trypanocidal efficacy against *Trypanosoma evansi*: new approach against diminazene aceturate resistance and toxicity. Exp. Parasitol..

[bib4] Banumathy G., Singh V., Pavithra S.R., Tatu U. (2003). Heat shock protein 90 function is essential for *Plasmodium falciparum* growth in human erythrocytes. J. Biol. Chem..

[bib5] Brayton K.A., Lau A.O.T., Herndon D.R., Hannick L., Kappmeyer L.S., Berens S.J. (2007). Genome sequence of *Babesia bovis* and comparative analysis of apicomplexan hemoprotozoa. PLoS Pathog..

[bib6] Chen B., Zhong D., Monteiro A. (2006). Comparative genomics and evolution of the HSP90 family of genes across all kingdoms of organisms. BMC Genom..

[bib7] Chou T.-C. (2006). Theoretical basis, experimental design, and computerized simulation of synergism and antagonism in drug combination studies. Pharmacol. Rev..

[bib8] Coldham N.G., Moore A.S., Dave M., Graham P.J., Sivapathasundaram S., Lake B.G., Sauer M.J. (1995). Imidocarb residues in edible bovine tissues and *in vitro* assessment of imidocarb metabolism and cytotoxicity. Drug Metab. Dispos..

[bib9] Echeverria P.C., Matrajt M., Harb O.S., Zappia M.P., Costas M.A., Roos D.S., Dubremetz J.F., Angel S.O. (2005). *Toxoplasma gondii* Hsp90 is a potential drug target whose expression and subcellular localization are developmentally regulated. J. Mol. Biol..

[bib10] Gillan V., O'Neill K., Maitland K., Sverdrup F.M., Devaney E. (2014). A repurposing strategy for Hsp90 inhibitors demonstrates their potency against filarial nematodes. PLoS Neglected Trop. Dis..

[bib11] Goo Y.K., Terkawi M.A., Jia H., Aboge G.O., Ooka H., Nelson B., Kim S., Sunaga F., Namikawa K., Igarashi I., Nishikawa Y., Xuan X. (2010). Artesunate, a potential drug for treatment of *Babesia* infection. Parasitol. Int..

[bib12] Guswanto A., Sivakumar T., Rizk M.A., Elsayed S.A.E., Youssef M.A., ElSaid E.E.S., Yokoyama N., Igarashi I. (2014). Evaluation of a fluorescence-based method for antibabesial drug screening. Antimicrob. Agents and Chemother.

[bib13] Igarashi I., Njonge F.K., Kaneko Y., Nakamura Y. (1998). *Babesia bigemina*: in vitro and in vivo effects of curdlan sulfate on the growth of parasites. Exp. Parasitol..

[bib14] Jilani K., Qadri S.M., Lang F. (2013). Geldanamycin-induced phosphatidylserine translocation in the erythrocyte membrane. Cell. Physiol. Biochem..

[bib15] Kim Y.S., Alarcon S.V., Lee S., Lee M.J., Giaccone G., Neckers L., Trepel J.B. (2009). Update on Hsp90 inhibitors in clinical trial. Curr. Top. Med. Chem..

[bib16] Kinnaird J.H., Singh M., Gillan V., Weir W., Calder E.D.D., Hostettler I., Tatu U., Devaney E., Shiels B.R. (2017). Characterization of HSP90 isoforms in transformed bovine leukocytes infected with *Theileria annulata*. Cell Microbiol..

[bib17] Korsinczky M., Chen N., Kotecka B., Saul A., Rieckmann K., Cheng Q. (2000). Mutations in *Plasmodium falciparum* cytochrome b that are associated with atovaquone resistance are located at a putative drug-binding site. Antimicrob. Agents and Chemother.

[bib18] Krause P.J., Lepore T., Sikand V.K., Gadbaw J., Burke G., Telford S.R., Brassard P., Pearl D., Azlanzadeh J., Christianson D., McGrath D., Spielman A. (2000). Atovaquone and azithromycin for the treatment of babesiosis. N. Engl. J. Med..

[bib19] Kumar N., Zhao Y., Graves P., Perez-Folgar J., Maloy L., Zheng H. (1990). Human immune response directed against *Plasmodium falciparum* heat shock-related proteins. Infect. Immun..

[bib20] Kuttler K.L. (1980). Pharmacotherapeutics of drugs used in treatment of anaplasmosis and babesiosis. J. Am. Vet. Med. Assoc..

[bib21] Levy M.G., Ristic M. (1980). *Babesia bovis*: continuous cultivation in a microaerophilous stationary phase culture. Science.

[bib22] Matsuu A., Miyamato K., Ikada H., Okano S., Higuchi S. (2006). Short report: cloning of the *Babesia gibsoni* cytochrome B gene and isolation of three single nucleotide polymorphisms from parasites present after atovaquone treatment. Am. J. Trop. Med.

[bib23] Meyer K.J., Shapiro T.A. (2013). Potent antitrypanosomal activities of heat shock protein 90 inhibitors *in vitro* and *in vivo*. J. Infect. Dis..

[bib24] Miyata Y., Nakamoto H., Neckers L. (2013). The therapeutic target Hsp90 and cancer hallmarks. Curr. Pharmaceut. Des..

[bib25] Mosqueda J., Olvera-Ramírez A., Aguilar-Tipacamú G., Cantó G. (2012). Current advances in detection and treatment of babesiosis. Curr. Med. Chem..

[bib26] Oliveira G.L.S., de Freitas R.M. (2015). Diminazene aceturate—an antiparasitic drug of antiquity: advances in pharmacology and therapeutics. Pharmacol. Res..

[bib27] Pallavi R., Roy N., Nageshan R.K., Talukdar P., Pavithra S.R., Reddy R., Venketesh S., Kumar R., Gupta A.K., Singh R.K., Yadav S.C., Tatu U. (2010). Heat shock protein 90 as a drug target against protozoan infections: biochemical characterization of Hsp90 from *Plasmodium falciparum* and *Trypanosoma evansi* and evaluation of its inhibitor as a candidate drug. J. Biol. Chem..

[bib28] Péroval M., Péry P., Labbé M. (2006). The heat shock protein 90 of *Eimeria tenella* is essential for invasion of host cell and schizont growth. Int. J. Parasitol..

[bib29] Pizarro J.C., Hills T., Senisterra G., Wernimont A.K., Mackenzie C., Norcross N.R., Ferguson M.A.J., Wyatt P.G., Gilbert I.H., Hui R. (2013). Exploring the *Trypanosoma brucei* Hsp83 potential as a target for structure guided drug design. PLoS Neglected Trop. Dis..

[bib30] Rajapakshage B.K.W., Yamasaki M., Hwang S.-J., Sasaki N., Murakami M., Tamura Y., Lim S.Y., Nakamura K., Ohta H., Takiguchi M. (2012). Involvement of mitochondrial genes of *Babesia gibsoni* in resistance to diminazene aceturate. J. Vet. Med. Sci..

[bib31] Rizk M.A., El-Sayed S.A.E.-S., AbouLaila M., Tuvshintulga B., Yokoyama N., Igarashi I. (2016). Large-scale drug screening against *Babesia divergens* parasite using a fluorescence-based high-throughput screening assay. Vet. Parasitol..

[bib32] Ruef B.J., Ward T.J., Oxner C.R., Conley P.G., Brown W.C., Rice-Ficht A.C. (2000). Phylogenetic analysis with newly characterized *Babesia bovis* hsp70 and hsp90 provides strong support for paraphyly within the piroplasms. Mol. Biochem. Parasitol..

[bib33] Suarez C.E., Noh S. (2011). Emerging perspectives in the research of bovine babesiosis and anaplasmosis. Vet. Parasitol..

[bib34] Vial H.J., Gorenflot A. (2006). Chemotherapy against babesiosis. Vet. Parasitol..

[bib35] Witola W., Tsuda A., Inoue N., Ohashi K., Onuma M. (2005). Acquired resistance to berenil in a cloned isolate of *Trypanosoma evansi* is associated with upregulation of a novel gene, TeDR40. Parasitology.

