# Supplementary material for: 17-DMAG inhibits the multiplication of several Babesia species and Theileria equi on in vitro cultures, and Babesia microti in mice
Source: Int J Parasitol Drugs Drug Resist. 2018 Mar 1;8(1):104–11. doi: 10.1016/j.ijpddr.2018.02.005 (PMC6114103; doi:10.1016/j.ijpddr.2018.02.005)
Supplement: Supplementary [file mmc1.pptx]

## Slide 1
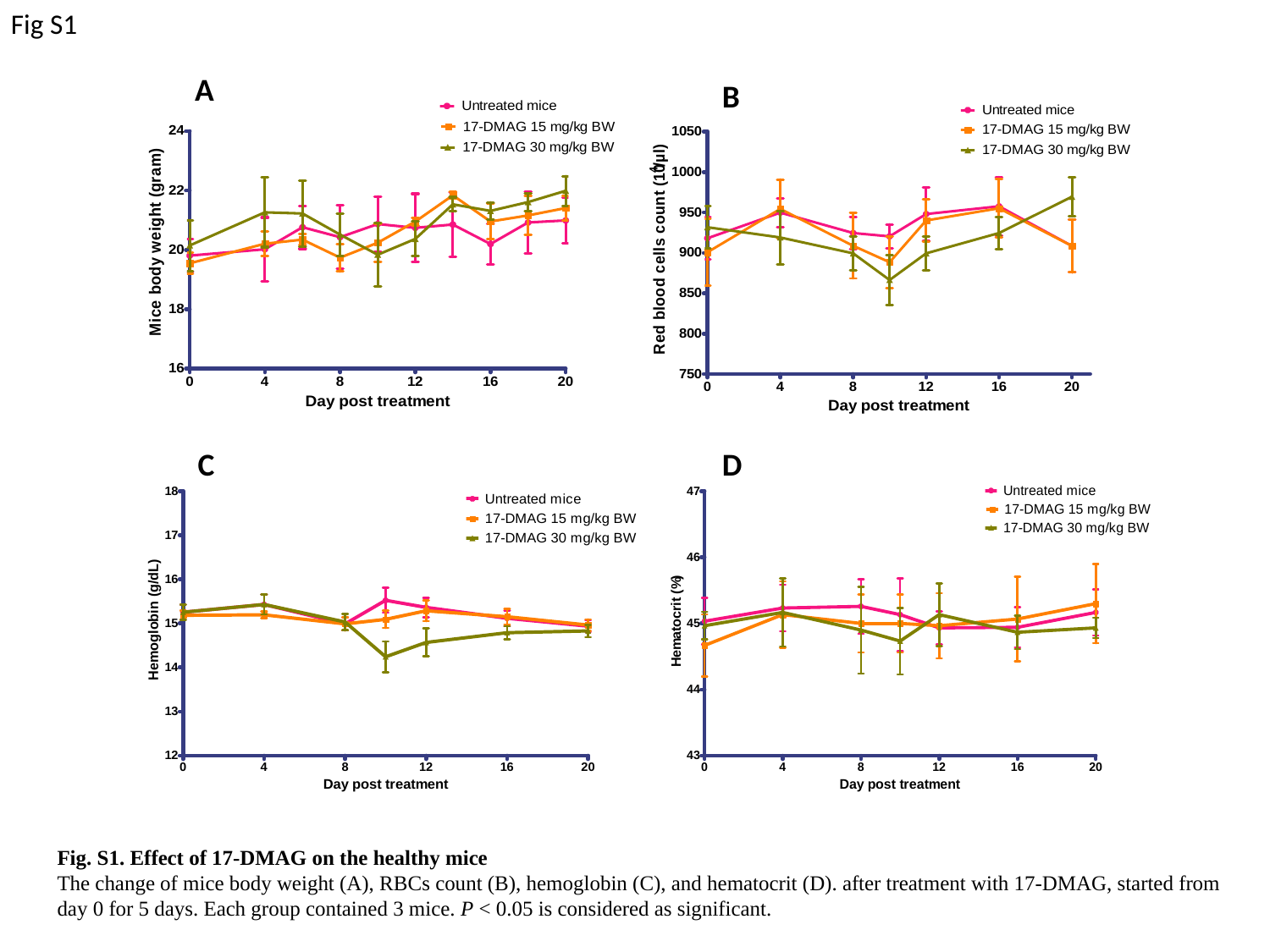

Fig S1
A
B
C
D
Fig. S1. Effect of 17-DMAG on the healthy mice
The change of mice body weight (A), RBCs count (B), hemoglobin (C), and hematocrit (D). after treatment with 17-DMAG, started from day 0 for 5 days. Each group contained 3 mice. P < 0.05 is considered as significant.

## Slide 2
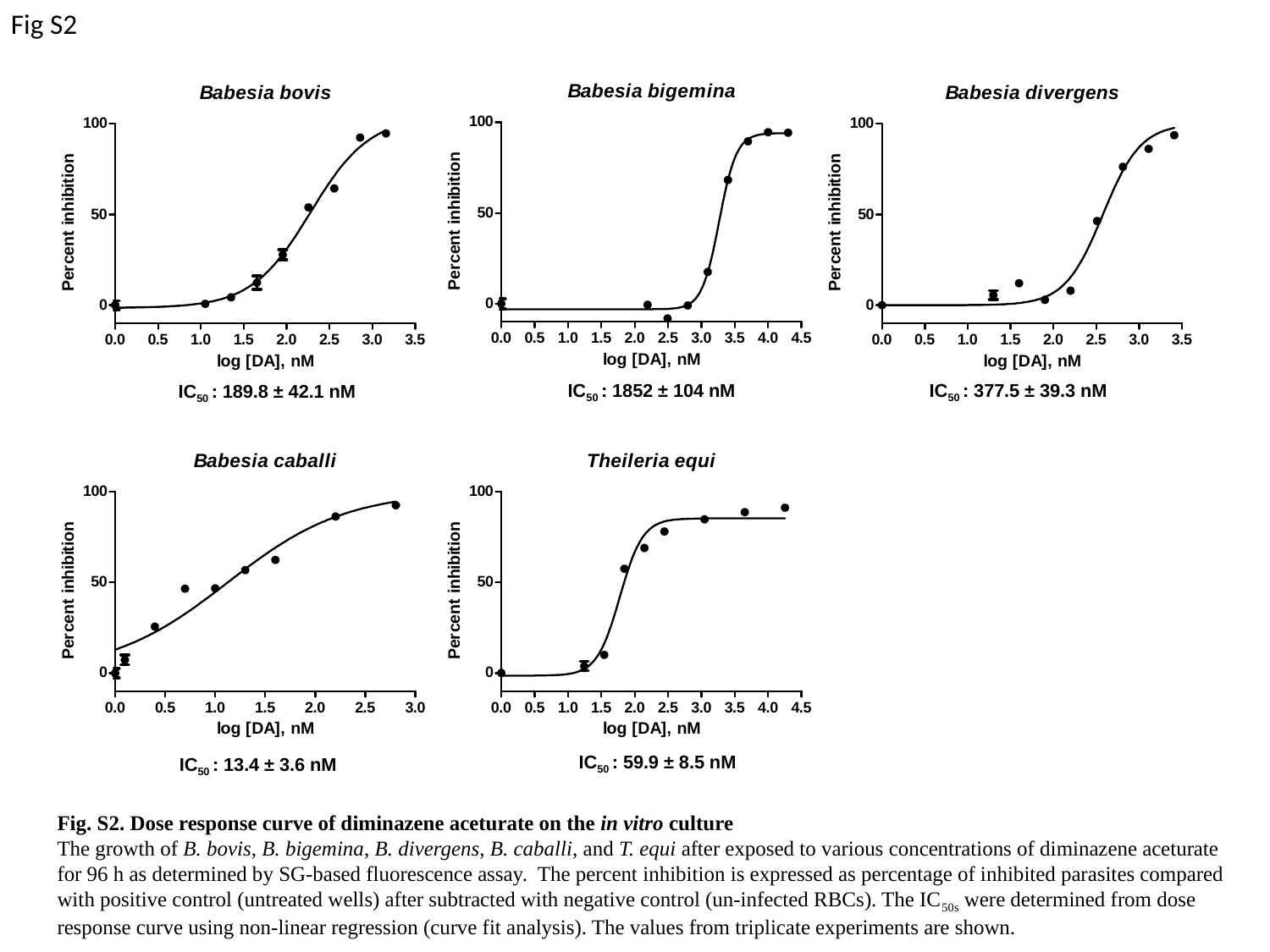

Fig S2
IC50 : 1852 ± 104 nM
IC50 : 377.5 ± 39.3 nM
IC50 : 189.8 ± 42.1 nM
IC50 : 59.9 ± 8.5 nM
IC50 : 13.4 ± 3.6 nM
Fig. S2. Dose response curve of diminazene aceturate on the in vitro culture
The growth of B. bovis, B. bigemina, B. divergens, B. caballi, and T. equi after exposed to various concentrations of diminazene aceturate for 96 h as determined by SG-based fluorescence assay. The percent inhibition is expressed as percentage of inhibited parasites compared with positive control (untreated wells) after subtracted with negative control (un-infected RBCs). The IC50s were determined from dose response curve using non-linear regression (curve fit analysis). The values from triplicate experiments are shown.

## Slide 3
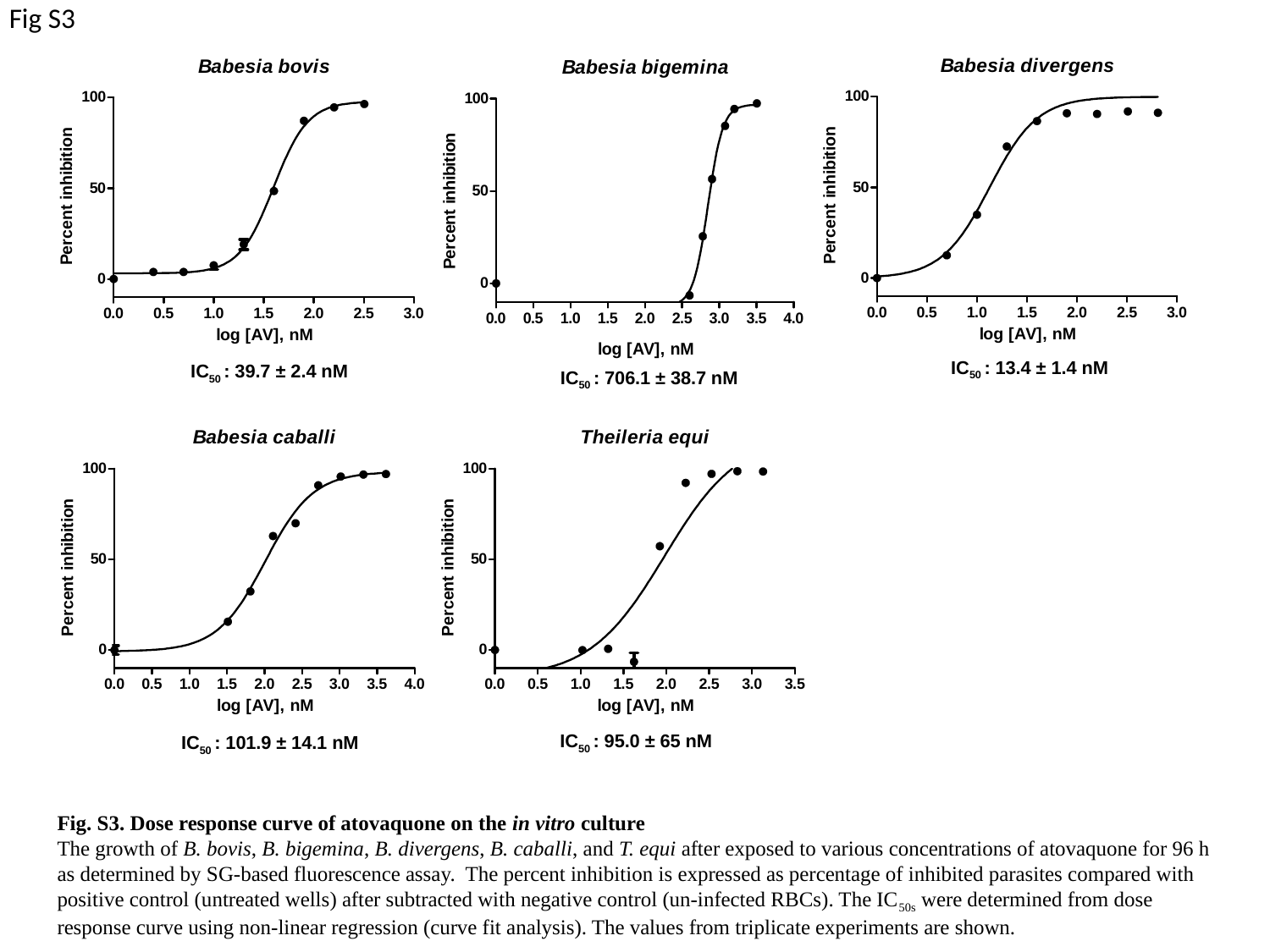

Fig S3
IC50 : 13.4 ± 1.4 nM
IC50 : 39.7 ± 2.4 nM
IC50 : 706.1 ± 38.7 nM
IC50 : 95.0 ± 65 nM
IC50 : 101.9 ± 14.1 nM
Fig. S3. Dose response curve of atovaquone on the in vitro culture
The growth of B. bovis, B. bigemina, B. divergens, B. caballi, and T. equi after exposed to various concentrations of atovaquone for 96 h as determined by SG-based fluorescence assay. The percent inhibition is expressed as percentage of inhibited parasites compared with positive control (untreated wells) after subtracted with negative control (un-infected RBCs). The IC50s were determined from dose response curve using non-linear regression (curve fit analysis). The values from triplicate experiments are shown.

## Slide 4
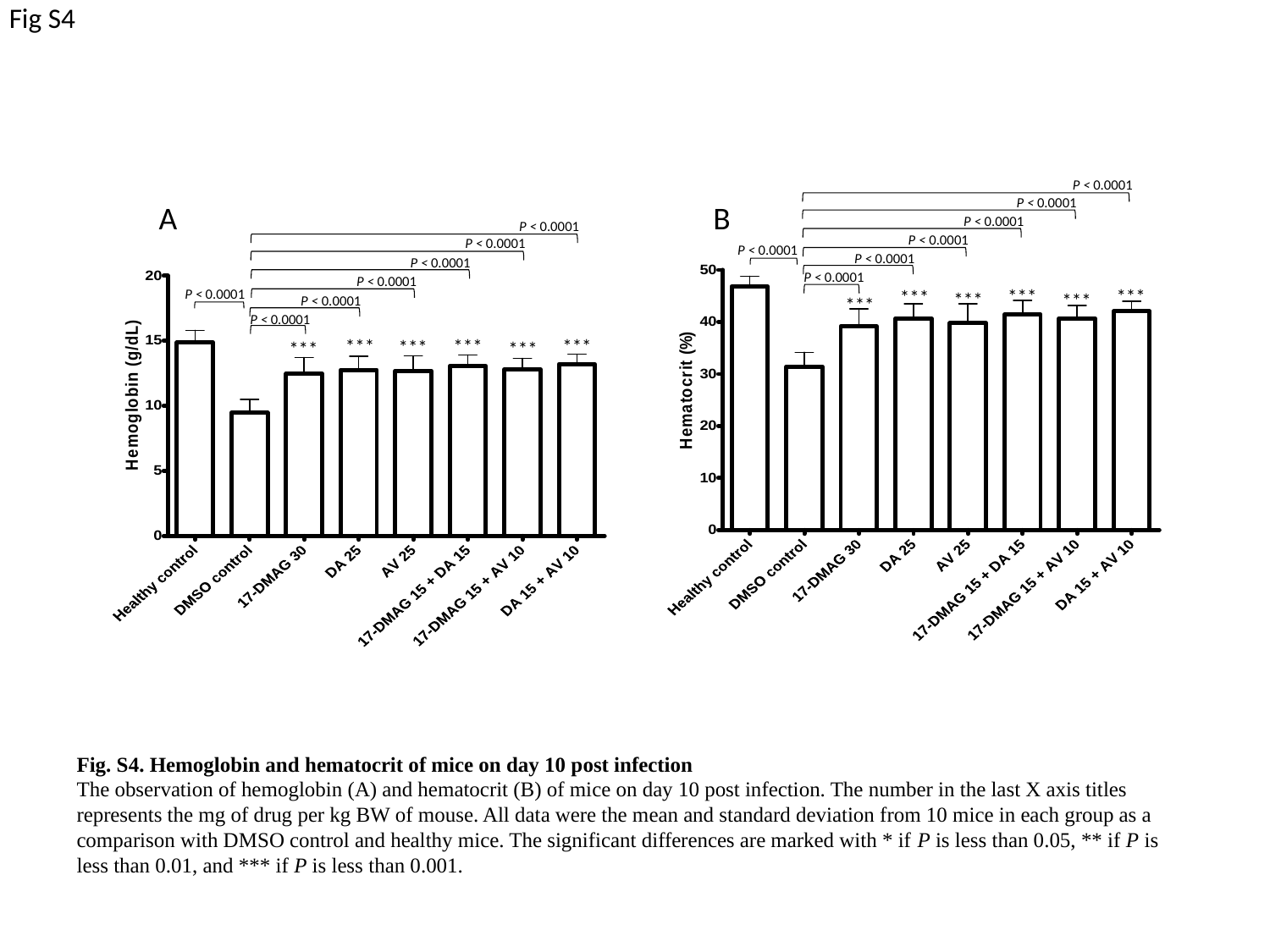

Fig S4
P < 0.0001
P < 0.0001
A
B
P < 0.0001
P < 0.0001
P < 0.0001
P < 0.0001
P < 0.0001
P < 0.0001
P < 0.0001
P < 0.0001
P < 0.0001
***
***
***
P < 0.0001
***
***
P < 0.0001
***
P < 0.0001
***
***
***
***
***
***
Fig. S4. Hemoglobin and hematocrit of mice on day 10 post infection
The observation of hemoglobin (A) and hematocrit (B) of mice on day 10 post infection. The number in the last X axis titles represents the mg of drug per kg BW of mouse. All data were the mean and standard deviation from 10 mice in each group as a comparison with DMSO control and healthy mice. The significant differences are marked with * if P is less than 0.05, ** if P is less than 0.01, and *** if P is less than 0.001.
